# Advances of Artificial Intelligence in Neuroimaging

**DOI:** 10.3390/brainsci15040351

**Published:** 2025-03-28

**Authors:** Iman Beheshti, Daichi Sone, Carson K. Leung

**Affiliations:** 1 Department of Human Anatomy and Cell Science, University of Manitoba, Winnipeg, MB R3E 0J9, Canada; 2 Department of Psychiatry, Jikei University School of Medicine, Tokyo 105-8461, Japan; daichisone@gmail.com; 3 Department of Computer Science, University of Manitoba, Winnipeg, MB R3T 2N2, Canada; carson.leung@umanitoba.ca

## 1. Introduction

Neuroimaging [1,2,3] is a rapidly evolving field that involves the use of non-invasive imaging techniques to visualize and study the structure and function of the human brain. This field has experienced transformative progress—as well as significant breakthroughs in terms of the accuracy, speed, and efficiency of identifying various brain disorders—over the past decade, largely driven by technological advancements and computational innovations. Among these, artificial intelligence (AI) has emerged as a pivotal tool, offering researchers and clinicians novel approaches to explore the brain’s structure and function [4,5,6,7,8,9,10]. AI models have been widely applied in the analysis and interpretation of neuroimaging data, aiding researchers and clinicians in diagnosing, treating, and monitoring patients with neurological and psychiatric disorders. This Special Issue, titled “Advances of AI in Neuroimaging”, was conceived to provide a platform for cutting-edge research at the intersection of AI and neuroimaging, aiming to revolutionize neuroscience and healthcare.

The primary motivation behind this Special Issue was the increasing demand for innovative solutions to address the complexity of neuroimaging data, especially in the context of neurological and psychiatric disorders. AI techniques, such as machine learning (ML) [11,12] and deep learning (DL) [13], offer unparalleled potential for biomarker discovery, disease prediction, and personalized treatment strategies. With the prevalence of brain disorders increasing, the need for accurate and efficient diagnostic tools is more pressing than ever.

This Special Issue sought to highlight both technical advancements and their practical implications for patient care and healthcare systems. The contributions span a range of neuroimaging modalities, including magnetic resonance imaging (MRI), positron emission tomography (PET), computed tomography (CT), and electroencephalography (EEG). By addressing challenges such as data complexity, model interpretability, and cost-efficiency, the featured research underscores the indispensable role of AI in advancing neuroimaging and its applications.

## 2. Summary of Accepted Papers

This Special Issue attracted widespread attention, receiving over 30 submissions from researchers from around the world. Each submission underwent rigorous quality control by the editorial team and the journal, ensuring adherence to the highest academic standards. The final selection of 17 accepted papers—consisting of 14 research articles, 1 review, 1 perspective, and 1 systematic review—represents cutting-edge research that successfully passed evaluations by expert peer reviewers in the field. Below is a synthesized overview of the published works.

Several studies (Contributions 1–3) focused on the application of ML and DL in medical imaging and surgical outcomes. For example, Ghanem et al. (Contribution 1) produced a *systematic review* examining the use of ML and DL models in predicting outcomes such as length of stay, readmissions, and mortality in spine surgery, revealing data imbalances and variations in evaluation metrics. Similarly, Rasheed et al. (Contribution 2) introduced a novel image enhancement methodology to improve the classification of brain tumors, achieving superior results compared with pre-trained models such as VGG16 and ResNet50, which are convolutional neural networks (CNNs) made up of 16 and 50 layers, respectively. The *review* by Shah and Heiss (Contribution 3) provided an in-depth look at AI’s applications in neurology, emphasizing its potential to predict neurological impairments, intracranial hemorrhage expansion, and outcomes for comatose patients, showcasing its diagnostic utility across diverse data sources.

Neuroimaging played a pivotal role in several contributions (Contributions 4–8). For instance, Rudroff (Contribution 4) provided his *perspective* on AI’s potential to analyze neuroimaging data, such as PET scans, to optimize treatment protocols and contribute to Long Coronavirus Disease (long COVID) research. Xiong et al. (Contribution 5) utilized support vector machines (SVMs) to classify Parkinson’s disease subtypes using arterial spin labeling MRI, while Wang et al. (Contribution 6) proposed a diagnostic model integrating multiple imaging modalities—namely, diffusion tensor imaging (DTI), structural MRI (sMRI), and functional MRI (fMRI)—to enhance the diagnosis of major depressive disorder (MDD). Similarly, Liu et al. (Contribution 7) introduced a low-rank tensor fusion algorithm to improve brain age estimation by integrating multimodal neuroimaging data, demonstrating enhanced accuracy. Yamao et al. (Contribution 8) proposed a deep learning method for directly predicting the centiloid scale based on amyloid PET images.

Several papers addressed neurodegenerative diseases and cognitive impairment (Contributions 9–12). For example, Saha et al. (Contribution 9) investigated baseline MRI data to predict the response of Alzheimer’s disease patients to repetitive transcranial magnetic stimulation (rTMS) treatment, while Grigas et al. (Contribution 10) demonstrated how super-resolved MRI images and optimized DL models improved mild cognitive impairment detection. Cerna et al. (Contribution 11) explored the neural mechanisms underlying Tai Chi’s benefits for cognitive and physical function, highlighting its potential to mitigate age-related declines in functional connectivity. Sone et al. (Contribution 12) examined disease progression patterns in temporal lobe epilepsy by using diffusion tensor imaging, revealing associations between white matter damage and clinical metrics.

Advancements in virtual reality (VR) and collaborative technologies also featured prominently. For instance, Tadayyoni et al. (Contribution 13) examined EEG data to classify user immersion in VR training environments, achieving high accuracy rates in distinguishing cognitive states and offering insights into real-time user engagement. Similarly, Shih et al. (Contribution 14) assessed inter-brain synchrony patterns in collaborative design tasks, comparing co-located and distributed settings to better understand team performance dynamics. This Special Issue also delves into cutting-edge methodologies, such as generative adversarial networks (GANs) (Contribution 15) and novel brain activity mapping (Contribution 16). Huynh et al. (Contribution 15) applied GANs to diagnose neurological conditions using functional connectivity data, while Huang (Contribution 16) introduced a method for analyzing task-evoked whole-brain activity, providing a unique lens to study individual brain variability during tasks. Lastly, Manabe et al. (Contribution 17) focused on skill assessment in laparoscopic surgery by comparing EEG-based models, revealing that a three-dimensional CNN approach significantly outperformed traditional methods in classifying expertise levels. This curated collection of papers underscores the transformative potential of AI-driven research in neuroimaging and its ability to address clinical and scientific challenges.

## 3. Statistics on the Special Issue

**Figure 1:**
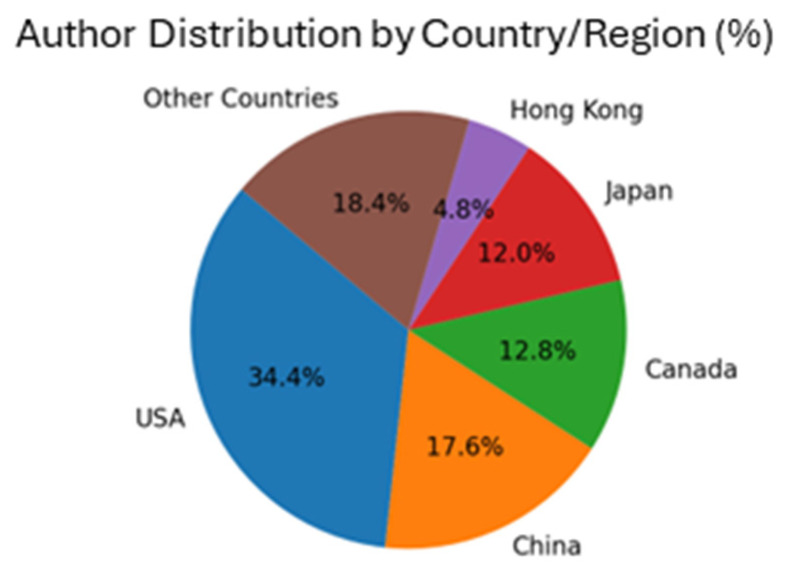
Geographic distribution of authors contributing to this Special Issue.

**Figure 2:**
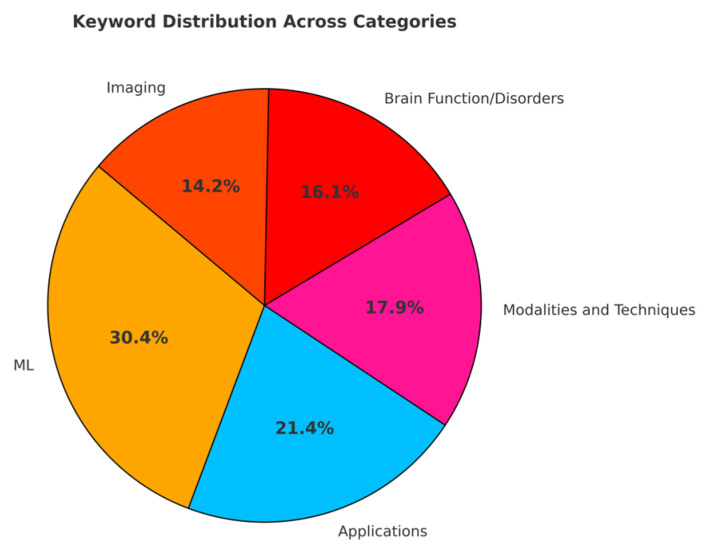
Distribution of keywords across research categories in this Special Issue.

The accepted papers were authored by 67 researchers from 14 countries, emphasizing the global collaboration underlying these advancements (Figure 1). Submissions were led by contributors from the USA (43 authors), China (22 authors), and Canada (16 authors), among others. The selected studies reflect diverse areas of expertise and applications, unified by their focus on leveraging AI to advance neuroimaging. The research featured in this Special Issue reflects prominent themes through its keywords: ML (17 keywords), neuroimaging techniques (8 keywords), brain functions and disorders (9 keywords), advanced methodologies (10 keywords), and practical applications (12 keywords) (Figure 2). Together, these works illustrate the breadth and depth of interdisciplinary innovation showcased in this Special Issue.

## 4. Conclusions

This Special Issue received significant attention, with the volume of submissions and the quality of accepted papers far exceeding our initial expectations. The rigorous selection process and peer review ensured that only the most impactful and innovative contributions were published. By highlighting the convergence of AI and neuroimaging, this issue lays the groundwork for future breakthroughs, fostering collaboration and advancing research at the intersection of neuroscience and technology.

## Abbreviations

The following abbreviations are used in this manuscript:

AI	Artificial intelligence
CNN	Convolutional neural network
COVID	Coronavirus disease
CT	Computed tomography
DL	Deep learning
DTI	Diffusion tensor imaging
EEG	Electroencephalography
fMRI	Functional magnetic resonance imaging
GAN	Generative adversarial network
MDD	Major depressive disorder
ML	Machine learning
MRI	Magnetic resonance imaging
PET	Positron emission tomography
rTMS	Repetitive transcranial magnetic stimulation
sMRI	Structural magnetic resonance imaging
SVMs	Support vector machines
VR	Virtual reality
